# Which indoor residual spraying insecticide best complements standard pyrethroid long-lasting insecticidal nets for improved control of pyrethroid resistant malaria vectors?

**DOI:** 10.1371/journal.pone.0245804

**Published:** 2021-01-28

**Authors:** Thomas Syme, Augustin Fongnikin, Damien Todjinou, Renaud Govoetchan, Martial Gbegbo, Mark Rowland, Martin Akogbeto, Corine Ngufor

**Affiliations:** 1 London School of Hygiene and Tropical Medicine (LSHTM), London, United Kingdom; 2 Centre de Recherches Entomologiques de Cotonou (CREC), Benin, West Africa; 3 Panafrican Malaria Vector Research Consortium (PAMVERC), Benin, West Africa; University of Crete, GREECE

## Abstract

**Background:**

Where resources are available, non-pyrethroid IRS can be deployed to complement standard pyrethroid LLINs with the aim of achieving improved vector control and managing insecticide resistance. The impact of the combination may however depend on the type of IRS insecticide deployed. Studies comparing combinations of pyrethroid LLINs with different types of non-pyrethroid IRS products will be necessary for decision making.

**Methods:**

The efficacy of combining a standard pyrethroid LLIN (DuraNet®) with IRS insecticides from three chemical classes (bendiocarb, chlorfenapyr and pirimiphos-methyl CS) was evaluated in an experimental hut trial against wild pyrethroid-resistant *Anopheles gambiae* s.l. in Cové, Benin. The combinations were also compared to each intervention alone. WHO cylinder and CDC bottle bioassays were performed to assess susceptibility of the local *An*. *gambiae* s.l. vector population at the Cové hut site to insecticides used in the combinations.

**Results:**

Susceptibility bioassays revealed that the vector population at Cové, was resistant to pyrethroids (<20% mortality) but susceptible to carbamates, chlorfenapyr and organophosphates (≥98% mortality). Mortality of wild free-flying pyrethroid resistant *An*. *gambiae* s.l. entering the hut with the untreated net control (4%) did not differ significantly from DuraNet® alone (8%, p = 0.169). Pirimiphos-methyl CS IRS induced the highest mortality both on its own (85%) and in combination with DuraNet® (81%). Mortality with the DuraNet® + chlorfenapyr IRS combination was significantly higher than each intervention alone (46% vs. 33% and 8%, p<0.05) demonstrating an additive effect. The DuraNet® + bendiocarb IRS combination induced significantly lower mortality compared to the other combinations (32%, p<0.05). Blood-feeding inhibition was very low with the IRS treatments alone (3–5%) but increased significantly when they were combined with DuraNet® (61% - 71%, p<0.05). Blood-feeding rates in the combinations were similar to the net alone. Adding bendiocarb IRS to DuraNet® induced significantly lower levels of mosquito feeding compared to adding chlorfenapyr IRS (28% vs. 37%, p = 0.015).

**Conclusions:**

Adding non-pyrethroid IRS to standard pyrethroid-only LLINs against a pyrethroid-resistant vector population which is susceptible to the IRS insecticide, can provide higher levels of vector mosquito control compared to the pyrethroid net alone or IRS alone. Adding pirimiphos-methyl CS IRS may provide substantial improvements in vector control while adding chlorfenapyr IRS can demonstrate an additive effect relative to both interventions alone. Adding bendiocarb IRS may show limited enhancements in vector control owing to its short residual effect.

## Background

Insecticide-treated nets (ITNs) and indoor residual spraying (IRS) are core interventions for preventing and controlling malaria [1]. Both methods have independently proven to be highly effective in reducing the burden of disease in diverse epidemiological settings. The substantial reductions in malaria morbidity and mortality observed in endemic countries over the last two decades, has been attributed to a significant increase in the roll out of ITNs and IRS during this period [2]. Where resources are available, both interventions have been deployed together in the same geographical location, with the primary aim of reducing the disease burden to a greater extent than could be achieved with either method alone [3,4].

The benefits of combining these interventions is, however, contentious. Some community-randomised controlled trials (RCTs) have compared epidemiological outcomes in communities receiving pyrethroid long-lasting insecticidal nets (LLINs) plus IRS versus LLINs alone, yielding conflicting results. Whilst trials in Tanzania [5] and Sudan [6] associated combined use of LLINs and IRS with significant reductions in malaria infection prevalence, similar studies in Benin [7], Eritrea [8], Ethiopia [9] and The Gambia [10] reported no such effect. Based on a Cochrane review demonstrating the inconsistencies in epidemiological evidence [4], the World Health Organisation (WHO) has recently issued a provisional recommendation against combining LLINs and IRS as a means of reducing malaria-associated morbidity and mortality [1]. National Malaria Control Programmes are encouraged to prioritise delivering either ITNs or IRS at high coverage and to a high standard, rather than introducing the second intervention as a means to compensate for deficiencies in the implementation of the first. However, this should not be interpreted to mean that the combined IRS and ITN approach is redundant in all settings. Considering the recent stall in progress against malaria [11] and increasing prevalence of pyrethroid resistance in malaria vector populations across Africa [12], combining interventions may be vital for high transmission settings and insecticide resistance management. Vector control programmes are expected to be guided by local evidence to decide when, where and how to combine IRS with ITNs for different epidemiological settings [1].

Evidence from RCTs and operational studies suggests that the impact of co-implementing LLINs and IRS may depend on a number of location-specific factors including: intervention coverage, transmission intensity, vector behaviour, choice of IRS insecticide and insecticide resistance [13,14]. The choice of IRS insecticide for a specific geographical setting whether deployed alone or together with LLINs will be influenced largely by the susceptibility of the target vector population to the insecticide, its mode of action, chemical properties and residual activity on wall substrates. To maximise the impact of the combined intervention approach, it is important to consider the interactions that may occur between the insecticide in the LLIN and the chosen IRS insecticide. Where the mode of action of the IRS insecticides is not complementary to the insecticide on the net, the combination might be redundant or result in lower levels of vector control than what is achievable with either intervention alone. Although pyrethroids are not recommended for IRS, the redundancy of combining pyrethroid IRS or wall linings with pyrethroid LLINs has been clearly demonstrated in experimental hut studies against pyrethroid-resistant malaria vectors in West Africa [15,16].

Insecticides belonging to four classes of compounds have been approved by the WHO for IRS against malaria vectors; pyrethroids, organophosphates, carbamates and more recently, the neonicotinoid clothianidin [1,17]. While RCTs are the most robust method for generating evidence on the impact of combining vector control interventions, they are often time-consuming, expensive and unrealistic in some settings. Evidence from experimental hut trials may provide some insights on what to expect when different types IRS insecticides are deployed to complement pyrethroid-only nets. In this study, we compared the impact of combining pyrethroid LLINs with IRS insecticides from three distinct chemical classes in experimental huts against wild free-flying pyrethroid-resistant malaria vectors in southern Benin.

## Methods

### Study site and experimental huts

The study was conducted at the CREC/LSHTM experimental hut station located in Covè, southern Benin (7°14′N2°18′E). The experimental huts are situated in a vast area of rice irrigation, which provide extensive and permanent breeding sites for mosquitoes. The rainy season extends from March to October and the dry season from November to February. *Anopheles coluzzii* and *An*. *gambiae* sensu stricto (s.s.) occur in sympatry, with the latter present at lower densities and predominantly in the dry season [18]. The vector population is susceptible to organophosphates and carbamates but exhibits intense resistance to pyrethroids (200-fold) [18]. Molecular genotyping and microarray studies have demonstrated a high frequency of the knockdown resistance L1014F allele (>90%) and overexpression of the cytochrome P450, CYP6P3, associated with pyrethroid detoxification [18]. The experimental huts used were of the West African design [19], made from concrete bricks with a corrugated iron roof and a ceiling with palm-thatch mats. Interior walls were cement plastered and each hut was constructed on a concrete base containing a water-filled moat to preclude predators. Entry of mosquitoes occurred via four window slits, 1 cm wide and 30 cm long, positioned on two sides of the hut. A wooden framed veranda made of polyethylene sheeting and screening mesh projected from the rear wall of each hut to capture exiting mosquitoes.

### Experimental hut treatments

The following treatments were assessed in 9 experimental huts:

1. Control (untreated hut)
2. Control (untreated net)
3. DuraNet® (Alpha-cypermethrin LLIN, 261 mg/m^2^, Shobikaa Impex)
4. Bendiocarb IRS applied at 400 mg/m^2^ (Ficam® WP, Bayer)
5. DuraNet® + Bendiocarb IRS applied at 400 mg/m^2^
6. Chlorfenapyr IRS applied at 250 mg/m^2^ (Sylando® 240SC, BASF)
7. DuraNet® + Chlorfenapyr IRS applied at 250 mg/m^2^
8. Pirimiphos-methyl CS IRS applied at 1000 mg/m^2^ (Actellic® 300CS, Syngenta)
9. DuraNet® + Pirimiphos-methyl CS IRS applied at 1000 mg/m^2^

Interior hut walls were sprayed from top to bottom with IRS insecticide solutions using a Hudson X-pert® compression sprayer equipped with a flat-fan nozzle. To improve spraying accuracy, spray swaths were pre-marked on hut walls and a guidance pole was attached to the end of the spray lance to maintain a fixed distance to the wall. After spraying each hut, the volume remaining in the spray tank was measured to assess the overall volume sprayed. All spray volumes were within 30% of the target. The palm thatch used on the ceiling was sprayed flat on the ground outside the experimental hut and allowed to dry for 2 hours prior to being fitted on the ceiling of the hut. Three replicate nets were used per LLIN treatment and these were rotated every 2 nights within the same hut. To simulate wear and tear from field use, 6 holes each measuring 16 cm^2^ were cut into each bed net (one per panel) used in the trial.

### Trial procedure

The trial began three days after IRS application and continued for 4 months, between July and October 2018 and followed WHO guidelines [19]. Nine (9) consenting human volunteers slept in experimental huts each trial night to attract mosquitoes. To account for bias due to differential attractiveness to mosquitoes, volunteers were rotated through experimental huts daily in accordance with a predetermined Latin square design. To mitigate the effect of hut position on mosquito entry, bed nets were rotated through experimental huts on a weekly basis. IRS treatments could not be rotated thus remained fixed throughout the trial. At dawn, sleepers collected all mosquitoes from under the net, the sleeping room and the veranda using a torch and aspirator and placed them in correspondingly labelled plastic cups. Mosquito collections were subsequently transferred to the laboratory for morphological identification and scoring of blood-feeding and mortality. To account for the slow-action of chlorfenapyr, delayed mortality was recorded every 24 h up to 72 h after collection for all treatments. All alive *An*. *gambiae* s.l. were held at 27 ± 2° C and 75 ± 10% RH and provided access to 10% (w/v) glucose solution.

### Outcome measures

The efficacy of each experimental hut treatment was expressed in terms of the following outcome measures:

- Exophily–exiting rates due to potential irritant effect of the treatment expressed as the proportion of mosquitoes collected in the veranda
- Deterrence–defined as the percentage reduction in numbers of mosquitoes collected in a treated hut relative to the untreated control
- Blood-feeding–proportion of blood-fed mosquitoes at the time of collection
- Blood-feeding inhibition–proportional reduction in blood-feeding in the treated hut relative to the untreated control. Calculated as follows:

$$BF\;inhibition\;(\%)=\frac{{100x(B}_{fu}-B_{ft})}{B_{fu}}$$

Where *B*_*fu*_ is the proportion of blood-fed mosquitoes in the untreated control and *B*_*ft*_ is the proportion of blood-fed mosquitoes in the treated hut.

- Personal protection (%)–reduction in the number of blood-fed mosquitoes in the treated hut relative to the untreated control. Calculated as follows:

$$Personal\;protection\;(\%)=\frac{{100x(B}_{u}-B_{t})}{B_{u}}$$

Where *B*_*u*_ is the number of blood-fed mosquitoes in the untreated control and *B*_*t*_ is the number of blood-fed mosquitoes in the huts with insecticide treatments.

- Mortality–proportion of dead mosquitoes after a 72 h holding period

Mosquito deterrence, blood-feeding inhibition and personal protection were calculated relative to the untreated net control hut for treatments which involved the LLIN and relative to the untreated hut control for IRS-only treatments.

### Residual activity of IRS treatments

Cone bioassays were performed at monthly intervals after spraying to assess the residual activity of IRS treatments on hut walls following WHO guidelines [19]. Laboratory-maintained, susceptible *An*. *gambiae* s.s. Kisumu mosquitoes were used for this purpose. In each IRS-treated hut, 50, 3–5-day old adult female mosquitoes were transferred to five cones attached to the walls and ceiling in batches of 10. As a control, mosquitoes were introduced into cones attached to an unsprayed wall. Mosquitoes were exposed to the surfaces for 30 mins before being transferred to netted cups. Mosquitoes were held at 27 ± 2° C and 75 ± 10% RH and provided access to 10% (w/v) glucose solution. Knockdown was recorded 1 h post-exposure and delayed mortality every 24 h up to 72 h thereafter.

### Susceptibility bioassays

WHO susceptibility cylinder bioassays [20] and CDC bottle bioassays [21] were performed in parallel to the experimental hut trial to determine the susceptibility of the vector population at the Covè experimental hut site to the constituent insecticides in the hut treatments. Adult F_1_ progeny of field-collected *An*. *gambiae* s.l. were exposed to filter papers treated with discriminating doses of alpha-cypermethrin (0.05%), permethrin (0.75%), bendiocarb (0.1%) and fenitrothion (1.0%) in WHO cylinders and CDC bottles impregnated with 100 μg of chlorfenapyr. Comparison was made with the laboratory-maintained, susceptible *An*. *gambiae* s.s. Kisumu colony. For each insecticide and strain, 100 adult female mosquitoes aged 2–3 days were introduced to four insecticide-treated cylinders/bottles in batches of 25. In the control arms, mosquitoes were exposed to silicone oil-treated filter papers and acetone-treated bottles. After 1 h exposure, mosquitoes were transferred to holding tubes and observation cups and provided access to 10% glucose solution (w/v). Mortality was recorded after 24 h for mosquitoes exposed to permethrin, alpha-cypermethrin, bendiocarb and fenitrothion in WHO cylinder bioassays and every 24 h up to 72 h for mosquitoes exposed to chlorfenapyr in CDC bottles. The insecticide treated filter papers used for the WHO cylinder bioassays were obtained from Universiti Sains Malaysia.

### Data analysis

Differences in proportional outcomes (exophily, blood-feeding, mortality) between experimental hut treatments were analysed using a blocked logistic regression model whereas differences in numerical outcomes (mosquito entry) were assessed using a negative binomial regression model. In addition to the fixed effect of the treatment, both models were adjusted to account for variation due to differential attractiveness of the volunteer sleepers and huts. Mortality in susceptibility bioassays was interpreted according to WHO criteria. All analyses were performed in Stata version 15.1.

### Ethical considerations

Ethical approval was obtained from the ethics review committees of the London School of Hygiene & Tropical Medicine (LSHTM) (Reference No. 15265) and the local Ministry of Health in Benin (Reference No. N°39/CEIC/CREC). All volunteers provided informed written consent and were offered a course of chemoprophylaxis (doxycycline) prior to their participation in the study and up to four weeks following its completion. A nurse was available throughout the study to assess any cases of fever. In the event a volunteer tested positive for malaria, they were immediately withdrawn from the trial and administered effective treatment.

## Results

### Susceptibility bioassay results

Mortality rates of wild *An*. *gambiae* s.l. from Covè in WHO susceptibility and CDC bottle bioassays are presented in Tables 1 and 2 respectively. Low proportions of adult F1 progeny of field-collected *An*. *gambiae* s.l. were killed following exposure to alpha-cypermethrin (11%) and permethrin (20%); demonstrating pyrethroid resistance in the vector population. In contrast, the vast majority if not all mosquitoes from Covè were killed following exposure to bendiocarb (98%), fenitrothion (100%) and chlorfenapyr (100%); demonstrating susceptibility to carbamates, organophosphates and chlorfenapyr. With the insecticide susceptible *An*. *gambiae* s.s. Kisumu colony, mortality was 100% with all insecticides.

**Table 1:** WHO susceptibility cylinder bioassay results with *Anopheles gambiae* sensu lato from Covè.

Treatment	Strain	N exposed	N dead	% Mortality	95% CI
Control	Kisumu	102	0	0	–
	Covè	102	0	0	–
Alpha-cypermethrin (0.05%)	Kisumu	102	102	100	–
	Covè	102	11	11	5–17
Permethrin (0.75%)	Kisumu	104	104	100	–
	Covè	105	21	20	12–28
Bendiocarb (0.1%)	Kisumu	104	104	100	–
	Covè	100	96	98	96–100
Fenitrothion (1%)	Kisumu	103	103	100	–
	Covè	101	101	100	–

**Table 2:** Susceptibility of field-collected *Anopheles gambiae* sensu lato from Covè to chlorfenapyr in CDC bottle bioassays.

Treatment	Strain	N exposed	N dead	% Mortality*	95% CI
Control	Kisumu	101	0	0	–
	Covè	100	0	0	–
Chlorfenapyr (100 μg)	Kisumu	103	103	100	–
	Covè	102	102	100	–

### Experimental hut results

#### Mosquito entry and exiting

A total of 4,698 female *An*. *gambiae* s.l. were collected in the experimental huts over the 4-month trial period (Tables 3 and S1). Due to the inability to rotate IRS, it was not possible to distinguish treatment-induced deterrence for the IRS treatments from differential attractiveness due to hut position. The proportion of mosquitoes exiting into the veranda for the untreated hut and untreated net controls were 45% and 39% respectively. Exiting was significantly higher in all experimental hut treatments relative to both controls (p<0.01), suggesting the insecticide treatments induced an irritant response which caused mosquitoes to seek refuge in the veranda. The highest level of mosquito exiting was achieved with bendiocarb IRS alone (77%). Adding bendiocarb IRS to DuraNet® also induced significantly higher levels of mosquito exiting compared to DuraNet® alone (57% with DuraNet® vs. 70% with DuraNet® + bendiocarb IRS, p = 0.001). This effect was, however, not observed with chlorfenapyr IRS and pirimiphos-methyl CS IRS; exiting rates were similar between the combinations with these IRS insecticides and DuraNet® alone, (59% - 61% vs 57%, p>0.05).

**Table 3:** Overall entry and exiting of wild, pyrethroid-resistant *Anopheles gambiae* sensu lato entering experimental huts in Covè, southern Benin.

Treatment	Total females caught	Average catch per night	% Deterrence	Total exiting	% Exophily*	95% CI
Untreated hut (control)	711	7	–	322	45^a^	42–49
Untreated net (control)	420	4	41	165	39^a^	35–44
DuraNet	619	6	13	350	57^b^	53–61
Bendiocarb IRS	508	5	29	389	77^c^	73–80
DuraNet + Bendiocarb IRS	537	6	25	373	70^cd^	66–73
Chlorfenapyr IRS	478	5	33	297	62^bd^	58–67
DuraNet + Chlorfenapyr IRS	411	4	42	241	59^b^	54–63
Pirimiphos-methyl CS IRS	528	6	26	356	67^d^	63–71
DuraNet + Pirimiphos-methyl CS IRS	486	5	32	298	61^bd^	57–66

*Values in the same column sharing a superscript letter do not differ significantly (p>0.05).

#### Blood-feeding and personal protection

Blood-feeding and personal protection results of wild, pyrethroid-resistant *An*. *gambiae* s.l. in the experimental huts are presented in Table 4. Blood-feeding rates with the untreated hut and untreated net controls were 95% and 55% respectively. DuraNet® induced significantly lower blood-feeding rates than the control untreated net (33% with DuraNet® vs. 55% with untreated net, p<0.001). Meanwhile, blood-feeding was high across all IRS treatments (91–92%) thus providing very limited blood-feeding inhibition (3% - 5%) compared to the untreated hut control (Table 4). All three combinations provided significantly higher levels of blood-feeding inhibition relative to the IRS treatments alone (60% - 71% vs. 3% - 5%, p<0.001). Between the combinations, DuraNet® + bendiocarb IRS induced the lowest blood-feeding rate (28%) and offered personal protection of 78% while DuraNet® + chlorfenapyr IRS induced a higher blood-feeding rate (37%, p = 0.015) though providing about the same levels of personal protection (77%).

**Table 4:** Blood-feeding results of wild, pyrethroid-resistant *Anopheles gambiae* sensu lato entering experimental huts in Covè, southern Benin.

Treatment	Total blood-fed	% Blood-fed*	95% CI	% Blood-feeding inhibition	% Personal protection
Untreated hut (control)	675	95^a^	93–97	–	–
Untreated net (control)	231	55^b^	50–60	42	66
DuraNet	202	33^cd^	29–36	66	70
Bendiocarb IRS	460	91^e^	88–93	5	32
DuraNet + Bendiocarb IRS	150	28^c^	24–32	71	78
Chlorfenapyr IRS	440	92^ae^	90–94	3	35
DuraNet + Chlorfenapyr IRS	153	37^d^	33–42	61	77
Pirimiphos-methyl CS IRS	488	92^ae^	90–95	3	28
DuraNet + Pirimiphos-methyl CS IRS	160	33^cd^	29–37	65	76

*Values in the same column sharing a superscript letter do not differ significantly (P>0.05).

#### Mortality rates in experimental huts

Overall mortality rates of wild, pyrethroid-resistant *An*. *gambiae* s.l. in the untreated hut and untreated net controls were 2% and 4% respectively (Table 5). Mortality with DuraNet® was low and did not differ significantly from the untreated net control (8% vs. 4%, p = 0.169). The IRS treatments alone and the combinations induced significantly higher mortality than DuraNet® and the untreated controls (p < 0.001); adding any IRS treatment onto DuraNet® therefore always significantly improved vector mortality compared to DuraNet® alone. Between the IRS only treatments, the highest mortality was achieved with pirimiphos-methyl CS (85%, p < 0.001) while chlorfenapyr and bendiocarb induced fairly similar mortality rates (34% and 28%, p = 0.09). Combining DuraNet® with bendiocarb and pirimiphos-methyl CS IRS treatments did not improve mortality compared to the corresponding IRS alone (bendiocarb: 28% vs. 32%, p = 0.265 and pirimiphos-methyl CS: 85% vs. 81%, p = 0.129) (Table 5). With chlorfenapyr IRS, mortality was significantly higher in the combination compared to chlorfenapyr IRS alone over the 4-month trial (34% vs. 46%, p < 0.001) (Fig 3). Between the combinations, the highest mortality was achieved with DuraNet® + pirimiphos-methyl CS IRS (81%). Mortality was significantly higher with the DuraNet® + chlorfenapyr IRS combination compared to DuraNet® + bendiocarb IRS (46% vs. 32%, p < 0.001) (Table 5).

**Table 5:** Overall mortality results of wild, pyrethroid-resistant *Anopheles gambiae* sensu lato entering experimental huts in Covè, southern Benin.

Treatment	Total dead	% Mortality*	95% CI
Untreated hut (control)	12	2^a^	1–3
Untreated net (control)	17	4^ab^	2–6
DuraNet	47	8^b^	6–10
Bendiocarb IRS	144	28^c^	24–32
DuraNet + Bendiocarb IRS	170	32^c^	28–36
Chlorfenapyr IRS	162	34^c^	30–38
DuraNet + Chlorfenapyr IRS	190	46^d^	41–51
Pirimiphos-methyl CS IRS	448	85^e^	82–88
DuraNet + Pirimiphos-methyl CS IRS	391	81^e^	77–84

*Values in the same column sharing a superscript letter do not differ significantly (P>0.05).

**Fig 1:**
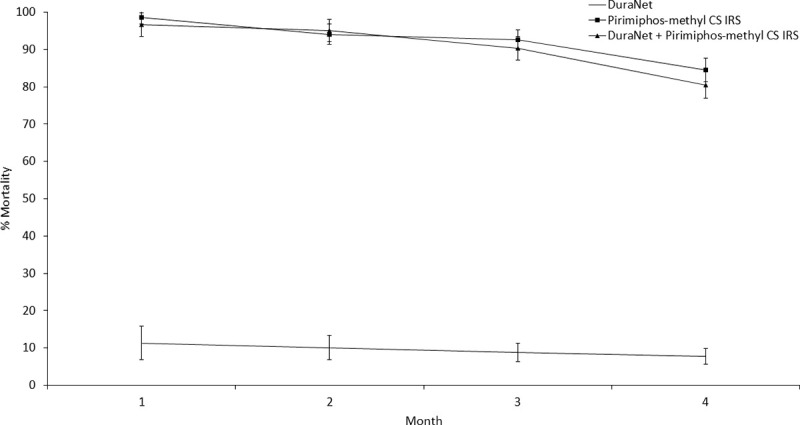
Monthly mortality rates of wild, free-flying pyrethroid-resistant *Anopheles gambiae* sensu lato entering experimental huts with pirimiphos-methyl CS IRS applied alone and in combination with DuraNet® in Covè, southern Benin. Mortality was cumulated over successive months. Error bars represent 95% CI.

**Fig 2:**
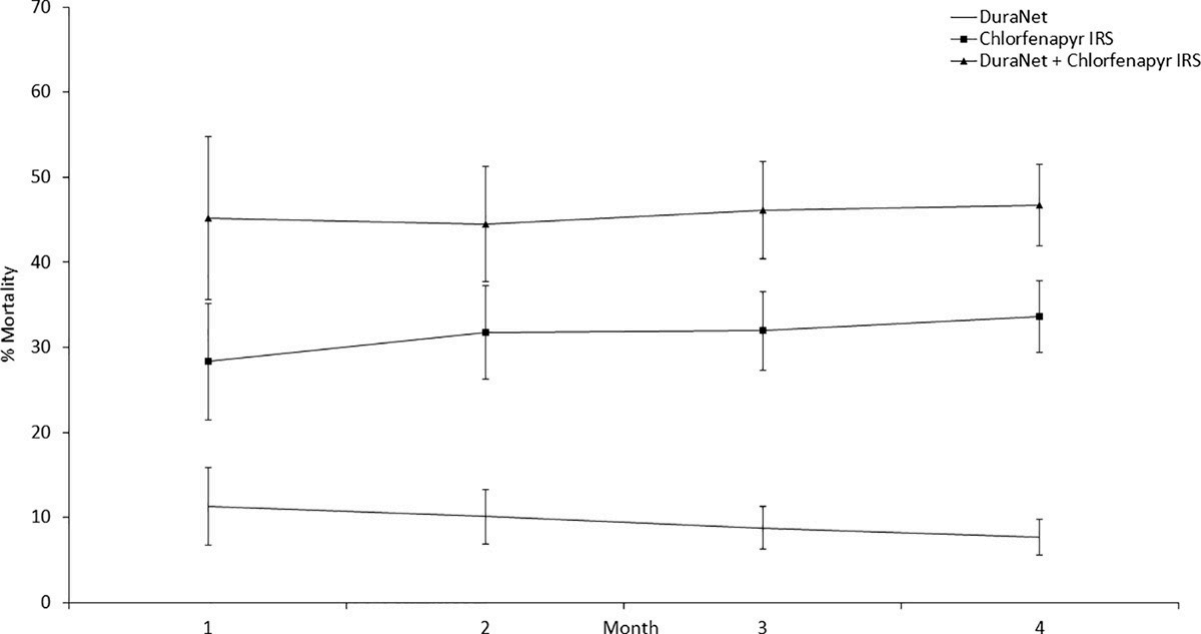
Monthly mortality rates of wild, free-flying pyrethroid-resistant *Anopheles gambiae* sensu lato entering experimental huts with chlorfenapyr IRS applied alone and in combination with DuraNet® in Covè, southern Benin. Mortality was cumulated over successive months. Error bars represent 95% CI.

**Fig 3:**
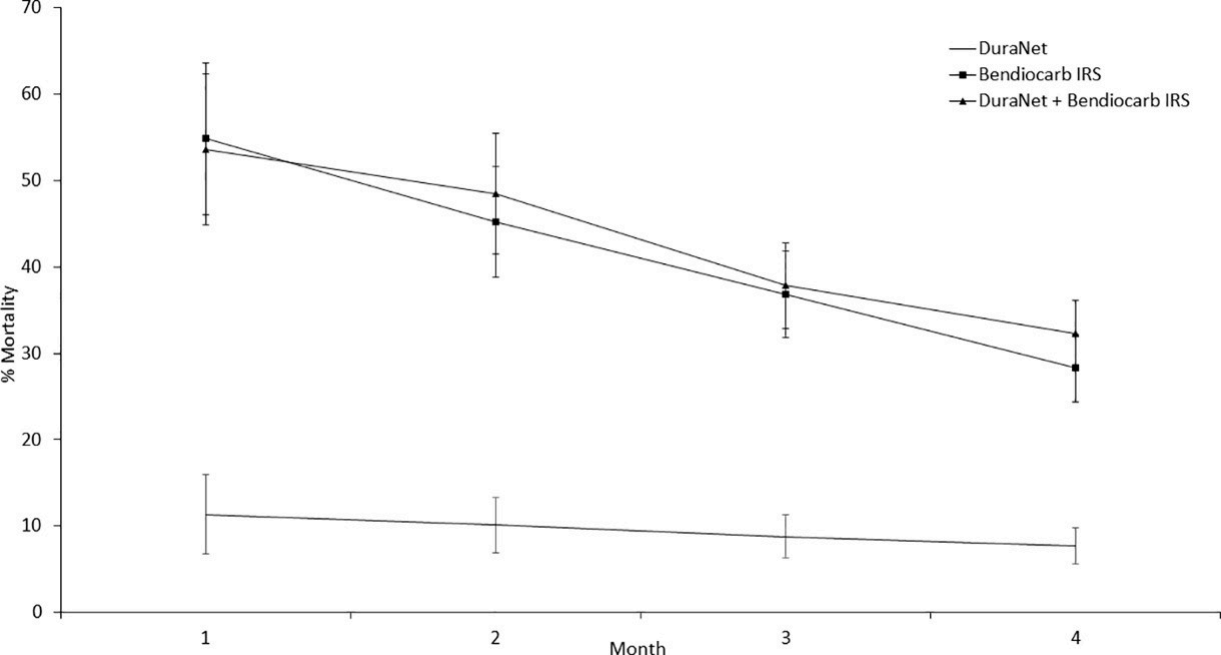
Monthly mortality rates of wild, free-flying pyrethroid-resistant *Anopheles gambiae* sensu lato entering experimental huts with bendiocarb IRS applied alone and in combination with DuraNet® in Covè, southern Benin. Mortality was cumulated over successive months. Error bars represent 95% CI.

Initial monthly wild mosquito mortality with pirimiphos-methyl CS IRS and chlorfenapyr IRS was steady and did not decline substantially over the course of the 4 months trial both when applied alone and in combination with DuraNet® (Figs 1 and 2). By contrast, wild mosquito mortality with bendiocarb IRS declined sharply from ~55% to about 28–32% by the last month of the trial both when applied alone and in combination with DuraNet® (Fig 3).

#### Wall cone bioassay results

**Fig 4:**
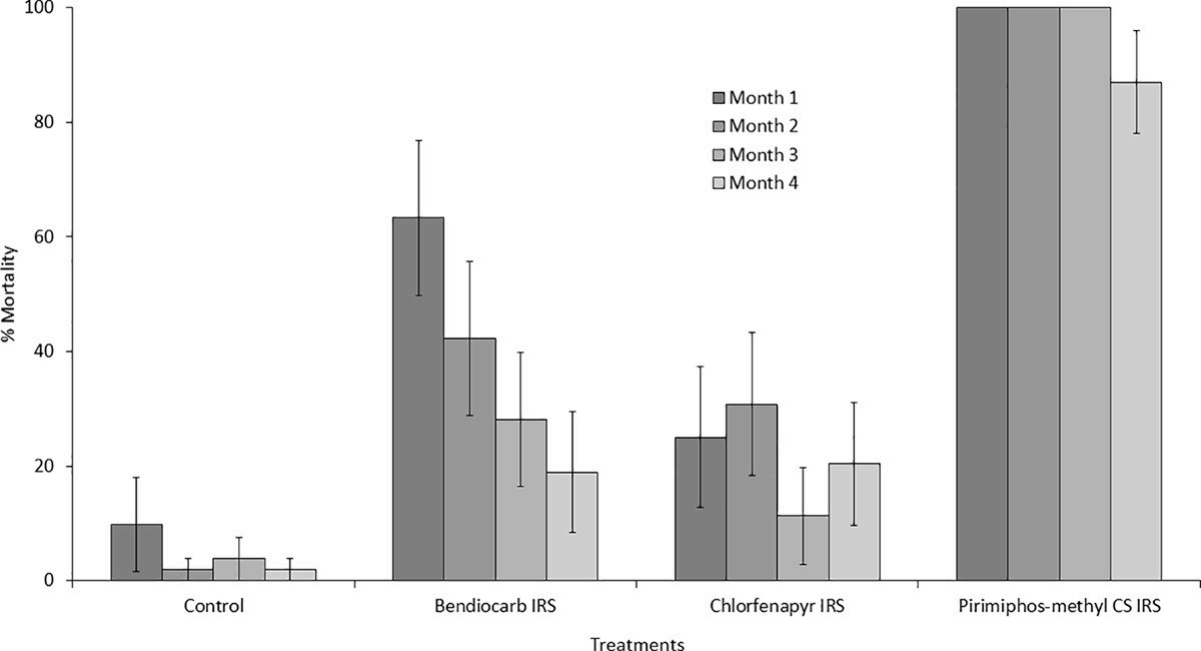
Mortality (72 h) of laboratory maintained, insecticide-susceptible *Anopheles gambiae* sensu stricto Kisumu colony exposed to IRS-treated hut walls in monthly, 30 mins wall cone bioassays in experimental huts in Covè, southern Benin. Error bars represent 95% CI. Approximately fifty (50) 2–3 days old mosquitoes were exposed for 30 mins to each IRS treated hut in batches of 10 per cone and mortality recorded after 72 h.

Mortality rates of the laboratory maintained, insecticide-susceptible *An*. *gambiae* s.s. Kisumu colony following exposure to IRS-treated walls in monthly, 30 mins wall cone bioassays are presented in Fig 4 with more details in supporting information (S2 Table). On pirimiphos-methyl CS-treated surfaces, cone bioassay mortality was very high (> 85%) throughout all 4 months of the trial. As observed with wild free-flying mosquitoes, a progressive decline in cone bioassay mortality with increasing months elapsed from spraying was observed with bendiocarb, beginning at 63% in month 1 and decreasing to 19% by month 4. Cone bioassay mortality with chlorfenapyr IRS was consistently low throughout the trial, ranging from 11–31%. Cone bioassay mortality on untreated control walls did not exceed 10% in any month of the trial (Fig 4).

## Discussion

The ultimate purpose of combining IRS with LLINs is to achieve greater levels of vector control through the differential effects of the interventions and modes of action of the insecticides involved than what is achievable with a single intervention on its own. Transmission dynamics models have suggested that the levels of vector mosquito mortality and blood-feeding inhibition observed in an experimental hut setting could be predictive of the capacity of an indoor vector control intervention or a combination of these to control vector populations, reduce vector biting and improve public health impact when deployed on a large scale [22,23]. In this study, we demonstrate that where vectors are resistant to pyrethroids but susceptible to a given non-pyrethroid IRS insecticide, it should be possible to achieve substantially improved vector control by adding the non-pyrethroid IRS to a standard pyrethroid-only LLIN to boost mortality rates through the IRS intervention, while benefiting from the blood-feeding inhibition provided by the LLIN.

The level of improved mortality achieved in the pyrethroid-only LLIN plus non-pyrethroid IRS combinations relative to the pyrethroid LLIN alone was clearly dependent on the type of non-pyrethroid IRS insecticide used. The combination with pirimiphos-methyl CS IRS induced the highest mortality rate (81%) and this can be attributed to the high and prolonged activity of the pirimiphos-methyl CS IRS formulation on cement walls as demonstrated in this study and previous studies in Benin [24]. This substantiates findings from community trials and operational studies demonstrating significant reductions in transmission of malaria by pyrethroid-resistant vector populations when a single round of pirimiphos-methyl CS IRS was deployed against a background of moderate to high coverage with standard pyrethroid nets [25–27]. By contrast the levels of mortality achieved in the combination with bendiocarb IRS was much lower (32%); attributable to the fast decline in efficacy with the insecticide as observed monthly in the experimental huts and in cone bioassays. The poor residual effect of bendiocarb for IRS is well documented [28–30]. The failure of a combination of bendiocarb treated walls and pyrethroid nets to provide improved control of clinical malaria compared to either intervention alone in a previous RCT in Benin [7] was also largely attributed to the short-lived efficacy of bendiocarb on the treated walls [13,14]. Multiple IRS campaign rounds may be necessary to achieve sustained levels of improved vector control when bendiocarb IRS is deployed to complement standard pyrethroid LLINs. Given the significant costs associated with running IRS campaigns, these findings raise questions over the suitability of currently available formulations of bendiocarb for IRS and the cost-effectiveness of its combination with pyrethroid nets. Considering the high initial mortality rates usually achieved with the insecticide, reformulation chemistry may help address the short residual efficacy of bendiocarb and improve the utility of this insecticide for vector control.

While mortality in the combinations with pirimiphos-methyl CS IRS and bendiocarb IRS were similar to each IRS alone, the combination with chlorfenapyr IRS induced significantly higher levels of mortality compared to chlorfenapyr IRS alone. This demonstrates an additive effect of the chlorfenapyr IRS plus pyrethroid LLINs combination which was not observed with the other IRS insecticides tested. This trend is consistent with previous experimental hut studies in Benin [31,32] though the levels of mortality achieved with the combination were substantially higher (73%–83% vs. 46%). The difference in mortality could be due to changes in the composition of the vector population and/or increasing pyrethroid resistance levels over time. Chlorfenapyr is a new repurposed pyrrole insecticide with a unique mode of action that has shown potential for IRS but often inducing moderate mortality rates when applied alone in experimental huts against pyrethroid-resistant malaria vectors [29,33]. It acts on the oxidative/metabolic pathway of insects hence its action can be enhanced by physical activity in the insect [34]. The additive mortality observed in the chlorfenapyr IRS plus pyrethroid net combination may therefore be attributed to the irritant effect of the pyrethroid in the LLIN on mosquitoes as they alight on the chlorfenapyr-treated wall after failed attempts to feed on the sleeper under the net. In addition, owing to the non-neurotoxic and non-irritant effect of chlorfenapyr [35], mosquitoes may have alighted on chlorfenapyr-treated walls for longer, leading to increased insecticide pick-up, potentially contributing to the additional mortality observed with this combination. The inability of the WHO 30-min cone bioassays to predict wild mosquito mortality with chlorfenapyr IRS in experimental huts [29,34] is further demonstrated in this study; while cone bioassay mortality of the susceptible Kisumu strain was very low, wild mosquito mortality was higher and consistent throughout the trial confirming previous findings [29]. Considering the prolonged effect of chlorfenapyr IRS on wild mosquitoes [29], substantial and sustained improvements in the control of pyrethroid-resistant vector populations can be expected if the IRS insecticide is deployed to complement pyrethroid LLINs.

IRS is highly effective for malaria vector control when deployed on its own but provides limited personal protection from mosquito biting and this is evident from the high blood-feeding rates observed with the IRS treatments alone. In the absence of a bed net, mosquitoes will usually feed on the human occupants in an IRS treated home before resting on the wall where they pick up the insecticide, consequently, direct blood-feeding inhibition is not expected when the intervention is applied independently. By contrast, pyrethroid LLINs reduce mosquito biting and provide substantial levels of personal protection for the user; an effect which is attributable to the physical barrier of the net and the excito-repellent effect of the pyrethroid insecticide. The findings from this study demonstrate that this effect can persist even against a vector population that has developed intense resistance to pyrethroids as observed in Covè [18]. By combining the IRS treatments with a pyrethroid LLIN, it was possible to achieve substantially improved levels of blood-feeding inhibition and personal protection compared to the IRS alone. The reduced exposure to infective bites constitutes a crucial advantage of the combination strategy over IRS alone. The blood-feeding rate in the combination appeared to depend on the type of IRS insecticide used albeit to a lesser extent than the levels of mortality achieved; lower blood-feeding rates and thus higher levels of blood-feeding inhibition were achieved in the combination with bendiocarb IRS compared to the combination with chlorfenapyr IRS. This can be due to differences in the mode of action of both IRS insecticides; in contrast to chlorfenapyr, bendiocarb acts on the insect’s nervous system eliciting an irritant rapid knockdown effect on mosquitoes [35] and this together with the excito-repellent effect of the pyrethroid in the standard pyrethroid LLIN could have contributed to the high levels of early exiting and reduced blood-feeding rates observed when both interventions were combined in a hut.

Overall, the results demonstrate the superiority of the organophosphate, pirimiphos-methyl CS for IRS on its own and in combination with pyrethroid LLINs over bendiocarb and the pyrrole, chlorfenapyr. However, this finding may not be generalisable to areas where vectors are resistant to organophosphates. Indeed, previous experimental hut studies in Cote d’Ivoire failed to demonstrate improved vector control with the combination compared to the net alone when standard pyrethroid LLINs were combined with pirimiphos-methyl CS-treated wall linings against a vector population that was resistant to pyrethroids and organophosphates [36]. Resistance to organophosphates and carbamates is unfortunately increasing in malaria vector populations especially in West Africa [37–40]. To mitigate its impact, vector control programmes are encouraged to rotate between IRS insecticides with different modes of action [41]. Considering that most IRS campaigns will usually be deployed against a background of high LLIN coverage, effective IRS rotation plans should prioritise IRS insecticides which in addition to providing improved and prolonged vector control, can efficiently complement LLINs. Based on the findings of this study, chlorfenapyr IRS shows some potential to be a useful addition to such IRS rotation plans in pyrethroid-resistant areas with high pyrethroid LLIN coverage. It will be interesting to investigate the impact of combining pyrethroid LLINs with other newly approved IRS formulations containing clothianidin [17].

The low mortality rates achieved with the standard pyrethroid net in this study (8%), further demonstrates the threat of pyrethroid resistance on the efficacy of standard pyrethroid nets. Pyrethroid resistance is widespread in malaria vectors across Africa and increasing in intensity the more they are used [12]. A new generation of LLINs treated with a pyrethroid and non-pyrethroid insecticide are now in advanced development. Studies have demonstrated the potential of some of these nets to provide improved control of clinical malaria transmitted by pyrethroid-resistant malaria vectors compared to standard pyrethroid nets [42–44]. As next generation nets come into the market, and are rolled out in endemic countries, it will be essential to explore any possible interactions with the different types of IRS insecticides that could be deployed together with them in a combined intervention approach.

## Conclusions

Adding non-pyrethroid IRS to standard pyrethroid-only nets against a pyrethroid-resistant vector population which was susceptible to the IRS insecticide, generally provided higher levels of vector mosquito control compared to the pyrethroid net alone or IRS alone; the IRS intervention was responsible for improved mosquito mortality while the pyrethroid net provided blood-feeding inhibition. The combination with pirimiphos-methyl CS IRS was the most efficacious. Adding chlorfenapyr IRS to the pyrethroid LLIN demonstrated an additive mortality effect relative to both interventions alone. The combination with bendiocarb IRS provided the lowest levels of mortality owing to its short residual effect but also induced lower levels of mosquito feeding compared to combinations with the other IRS insecticides.

## Supporting information

S1 Table Overall experimental hut results with wild free-flying pyrethroid resistant *An gambiae* s.l. in Cove, Benin. (XLSX)

S2 Table Detailed in situ cone bioassays data with susceptible *An gambiae* Kisumu in experimental huts. (XLSX)

## References

[1] WHO. Guidelines for malaria vector control. Geneva, Switzerland: World Health Organization 2019. 30844152

[2] Tangena J-AA, Hendriks CMJ, Devine M, Tammaro M, Trett AE, Williams I, et al Indoor residual spraying for malaria control in sub-Saharan Africa 1997 to 2017: an adjusted retrospective analysis. Malaria Journal. 2020;19(1):150 10.1186/s12936-020-03216-6 32276585 PMC7149868

[3] Kleinschmidt I, Schwabe C, Shiva M, Segura JL, Sima V, Mabunda SJ, et al Combining indoor residual spraying and insecticide-treated net interventions. Am J Trop Med Hyg. 2009;81(3):519–24. Epub 2009/08/27. 19706925 PMC3836236

[4] Choi L, Pryce J, Garner P. Indoor residual spraying for preventing malaria in communities using insecticide‐treated nets. Cochrane Database of Systematic Reviews. 2019;(5). 10.1002/14651858.CD012688.pub2 . 31120132 PMC6532761

[5] Protopopoff N, Wright A, West PA, Tigererwa R, Mosha FW, Kisinza W, et al Combination of Insecticide Treated Nets and Indoor Residual Spraying in Northern Tanzania Provides Additional Reduction in Vector Population Density and Malaria Transmission Rates Compared to Insecticide Treated Nets Alone: A Randomised Control Trial. PLoS One. 2015;10(11):e0142671 Epub 2015/11/17. 10.1371/journal.pone.0142671 26569492 PMC4646432

[6] Kafy HT, Ismail BA, Mnzava AP, Lines J, Abdin MSE, Eltaher JS, et al Impact of insecticide resistance in Anopheles arabiensis on malaria incidence and prevalence in Sudan and the costs of mitigation. Proc Natl Acad Sci U S A. 2017;114(52):E11267–E75. Epub 2017/12/13. 10.1073/pnas.1713814114 29229808 PMC5748194

[7] Corbel V, Akogbeto M, Damien GB, Djenontin A, Chandre F, Rogier C, et al Combination of malaria vector control interventions in pyrethroid resistance area in Benin: a cluster randomised controlled trial. Lancet Infect Dis. 2012;12(8):617–26. Epub 2012/06/12. 10.1016/S1473-3099(12)70081-6 . 22682536

[8] Keating J, Locatelli A, Gebremichael A, Ghebremeskel T, Mufunda J, Mihreteab S, et al Evaluating indoor residual spray for reducing malaria infection prevalence in Eritrea: results from a community randomized control trial. Acta Trop. 2011;119(2–3):107–13. Epub 2011/05/14. 10.1016/j.actatropica.2011.04.015 . 21565149

[9] Loha E, Deressa W, Gari T, Balkew M, Kenea O, Solomon T, et al Long-lasting insecticidal nets and indoor residual spraying may not be sufficient to eliminate malaria in a low malaria incidence area: results from a cluster randomized controlled trial in Ethiopia. Malar J. 2019;18(1):141 Epub 2019/04/20. 10.1186/s12936-019-2775-1 30999957 PMC6471954

[10] Pinder M, Jawara M, Jarju LB, Salami K, Jeffries D, Adiamoh M, et al Efficacy of indoor residual spraying with dichlorodiphenyltrichloroethane against malaria in Gambian communities with high usage of long-lasting insecticidal mosquito nets: a cluster-randomised controlled trial. Lancet. 2015;385(9976):1436–46. Epub 2014/12/17. 10.1016/S0140-6736(14)61007-2 . 25498847

[11] WHO. World Malaria Report 2020. Geneva, Switzerland: World Health Organisation 2020.

[12] WHO. Global report on insecticide resistance in malaria vectors: 2010–2016. Geneva, Switzerland: World Health Organization 2018.

[13] Lines J, Kleinschmidt I. Combining malaria vector control interventions: some trial design issues. Pathog Glob Health. 2013;107(1):1–4. Epub 2013/02/26. 10.1179/2047772413Z.000000000104 23432856 PMC4001595

[14] WHO. Review of current evidence on combining indoor residual spraying and long-lasting insecticidal nets. World Health Organisation 2014;https://www.who.int/malaria/mpac/background-combining-irs-llins-mar2014.pdf?ua=1.

[15] Ngufor C, Tchicaya E, Koudou B, N'Fale S, Dabire R, Johnson P, et al Combining organophosphate treated wall linings and long-lasting insecticidal nets for improved control of pyrethroid resistant Anopheles gambiae. PLoS One. 2014;9(1):e83897 Epub 2014/01/11. 10.1371/journal.pone.0083897 24409286 PMC3883662

[16] Chandre F, Dabire RK, Hougard J-M, Djogbenou LS, Irish SR, Rowland M, et al Field efficacy of pyrethroid treated plastic sheeting (durable lining) in combination with long lasting insecticidal nets against malaria vectors. Parasites & Vectors. 2010;3(1):65 10.1186/1756-3305-3-65 20682050 PMC2920241

[17] WHO. List of WHO prequalified vector control products. Geneva, Switzerland: World Health Organization 2020;https://www.who.int/pq-vector-control/prequalified-lists/PrequalifiedProducts27January2020.pdf?ua=1.

[18] Ngufor C, N'Guessan R, Fagbohoun J, Subramaniam K, Odjo A, Fongnikin A, et al Insecticide resistance profile of Anopheles gambiae from a phase II field station in Cove, southern Benin: implications for the evaluation of novel vector control products. Malar J. 2015;14:464 Epub 2015/11/20. 10.1186/s12936-015-0981-z 26581678 PMC4652434

[19] WHO. Guidelines for testing mosquito adulticides for indoor residual spraying and treatment of mosquito nets. World Health Organisation 2006;Geneva, Switzerland.

[20] WHO. Test procedures for insecticide resistance monitoring in malaria vector mosquitoes; second edition World Health Organisation 2016.

[21] Brogdon W, Chan A. Guideline for evaluating insecticide resistance in vectors using the CDC bottle bioassay. USA: CDC Atlanta 2010.

[22] Churcher TS, Lissenden N, Griffin JT, Worrall E, Ranson H. The impact of pyrethroid resistance on the efficacy and effectiveness of bednets for malaria control in Africa. Elife. 2016;5 Epub 2016/08/23. 10.7554/eLife.16090 27547988 PMC5025277

[23] Sherrard-Smith E, Griffin JT, Winskill P, Corbel V, Pennetier C, Djenontin A, et al Systematic review of indoor residual spray efficacy and effectiveness against Plasmodium falciparum in Africa. Nat Commun. 2018;9(1):4982 Epub 2018/11/28. 10.1038/s41467-018-07357-w 30478327 PMC6255894

[24] Rowland M, Boko P, Odjo A, Asidi A, Akogbeto M, N'Guessan R. A new long-lasting indoor residual formulation of the organophosphate insecticide pirimiphos methyl for prolonged control of pyrethroid-resistant mosquitoes: an experimental hut trial in Benin. PLoS One. 2013;8(7):e69516 Epub 2013/08/13. 10.1371/journal.pone.0069516 23936033 PMC3720653

[25] Abong’o B, Gimnig JE, Torr SJ, Longman B, Omoke D, Muchoki M, et al Impact of indoor residual spraying with pirimiphos-methyl (Actellic 300CS) on entomological indicators of transmission and malaria case burden in Migori County, western Kenya. Scientific Reports. 2020;10(1):4518 10.1038/s41598-020-61350-2 32161302 PMC7066154

[26] Salako AS, Dagnon F, Sovi A, Padonou GG, Aïkpon R, Ahogni I, et al Efficacy of Actellic 300 CS-based indoor residual spraying on key entomological indicators of malaria transmission in Alibori and Donga, two regions of northern Benin. Parasites & Vectors. 2019;12(1):612 10.1186/s13071-019-3865-1 31888730 PMC6937814

[27] Tugume A, Muneza F, Oporia F, Kiconco A, Kihembo C, Kisakye AN, et al Effects and factors associated with indoor residual spraying with Actellic 300 CS on malaria morbidity in Lira District, Northern Uganda. Malaria Journal. 2019;18(1):44 10.1186/s12936-019-2681-6 30791906 PMC6383239

[28] Yeebiyo Y, Dengela D, Tesfaye AG, Anshebo GY, Kolyada L, Wirtz R, et al Short persistence of bendiocarb sprayed on pervious walls and its implication for the indoor residual spray program in Ethiopia. Parasit Vectors. 2016;9:266 Epub 2016/05/07. 10.1186/s13071-016-1549-7 27151229 PMC4858853

[29] Ngufor C, Fongnikin A, Hobbs N, Gbegbo M, Kiki L, Odjo A, et al Indoor spraying with chlorfenapyr (a pyrrole insecticide) provides residual control of pyrethroid-resistant malaria vectors in southern Benin. Malaria Journal. 2020;19(1):249 10.1186/s12936-020-03325-2 32660479 PMC7359555

[30] Djènontin A, Aïmihouè O, Sèzonlin M, Damien GB, Ossè R, Soukou B, et al The residual life of bendiocarb on different substrates under laboratory and field conditions in Benin, Western Africa. BMC Research Notes. 2013;6(1):458 10.1186/1756-0500-6-458 24220151 PMC3831871

[31] Ngufor C, N'Guessan R, Boko P, Odjo A, Vigninou E, Asidi A, et al Combining indoor residual spraying with chlorfenapyr and long-lasting insecticidal bed nets for improved control of pyrethroid-resistant Anopheles gambiae: an experimental hut trial in Benin. Malar J. 2011;10:343 Epub 2011/11/18. 10.1186/1475-2875-10-343 22087506 PMC3229591

[32] Ngufor C, Fagbohoun J, Critchley J, N'Guessan R, Todjinou D, Malone D, et al Which intervention is better for malaria vector control: insecticide mixture long-lasting insecticidal nets or standard pyrethroid nets combined with indoor residual spraying? Malar J. 2017;16(1):340 Epub 2017/08/18. 10.1186/s12936-017-1987-5 28814307 PMC5559808

[33] Ngufor C, Critchley J, Fagbohoun J, N'Guessan R, Todjinou D, Rowland M. Chlorfenapyr (A Pyrrole Insecticide) Applied Alone or as a Mixture with Alpha-Cypermethrin for Indoor Residual Spraying against Pyrethroid Resistant Anopheles gambiae sl: An Experimental Hut Study in Cove, Benin. PLoS One. 2016;11(9):e0162210 Epub 2016/09/03. 10.1371/journal.pone.0162210 27588945 PMC5010291

[34] Oxborough RM, N'Guessan R, Jones R, Kitau J, Ngufor C, Malone D, et al The activity of the pyrrole insecticide chlorfenapyr in mosquito bioassay: towards a more rational testing and screening of non-neurotoxic insecticides for malaria vector control. Malar J. 2015;14:124 Epub 2015/04/17. 10.1186/s12936-015-0639-x 25879231 PMC4390098

[35] Achee NL, Sardelis MR, Dusfour I, Chauhan KR, Grieco JP. Characterization of spatial repellent, contact irritant, and toxicant chemical actions of standard vector control compounds. J Am Mosq Control Assoc. 2009;25(2):156–67. Epub 2009/08/06. 10.2987/08-5831.1 . 19653497

[36] Ngufor C, Chouaibou M, Tchicaya E, Loukou B, Kesse N, N'Guessan R, et al Combining organophosphate-treated wall linings and long-lasting insecticidal nets fails to provide additional control over long-lasting insecticidal nets alone against multiple insecticide-resistant Anopheles gambiae in Cote d'Ivoire: an experimental hut trial. Malar J. 2014;13:396 Epub 2014/10/11. 10.1186/1475-2875-13-396 25301219 PMC4203900

[37] Ahoua Alou LP, Koffi AA, Adja MA, Tia E, Kouassi PK, Koné M, et al Distribution of ace-1Rand resistance to carbamates and organophosphates in Anopheles gambiae s.s. populations from Côte d'Ivoire. Malaria Journal. 2010;9(1):167 10.1186/1475-2875-9-167 20553593 PMC2908637

[38] Elanga-Ndille E, Nouage L, Ndo C, Binyang A, Assatse T, Nguiffo-Nguete D, et al The G119S Acetylcholinesterase (Ace-1) Target Site Mutation Confers Carbamate Resistance in the Major Malaria Vector Anopheles gambiae from Cameroon: A Challenge for the Coming IRS Implementation. Genes (Basel). 2019;10(10). Epub 2019/10/17. 10.3390/genes10100790 31614683 PMC6826778

[39] Edi CV, Koudou BG, Jones CM, Weetman D, Ranson H. Multiple-insecticide resistance in Anopheles gambiae mosquitoes, Southern Cote d'Ivoire. Emerg Infect Dis. 2012;18(9):1508–11. Epub 2012/08/31. 10.3201/eid1809.120262 22932478 PMC3437712

[40] Dabire KR, Diabate A, Namontougou M, Djogbenou L, Kengne P, Simard F, et al Distribution of insensitive acetylcholinesterase (ace-1R) in Anopheles gambiae s.l. populations from Burkina Faso (West Africa). Trop Med Int Health. 2009;14(4):396–403. Epub 2009/03/04. 10.1111/j.1365-3156.2009.02243.x . 19254231

[41] WHO. Global Plan for Insecticide Resistance Management in Malaria Vectors. Geneva, Switzerland: World Health Organization 2012.

[42] Protopopoff N, Mosha JF, Lukole E, Charlwood JD, Wright A, Mwalimu CD, et al Effectiveness of a long-lasting piperonyl butoxide-treated insecticidal net and indoor residual spray interventions, separately and together, against malaria transmitted by pyrethroid-resistant mosquitoes: a cluster, randomised controlled, two-by-two factorial design trial. Lancet. 2018;391(10130):1577–88. Epub 2018/04/16. 10.1016/S0140-6736(18)30427-6 29655496 PMC5910376

[43] Ngufor C, Agbevo A, Fagbohoun J, Fongnikin A, Rowland M. Efficacy of Royal Guard, a new alpha-cypermethrin and pyriproxyfen treated mosquito net, against pyrethroid-resistant malaria vectors. Scientific Reports. 2020;10(1):12227 10.1038/s41598-020-69109-5 32699237 PMC7376134

[44] N'Guessan R, Odjo A, Ngufor C, Malone D, Rowland M. A Chlorfenapyr Mixture Net Interceptor(R) G2 Shows High Efficacy and Wash Durability against Resistant Mosquitoes in West Africa. PLoS One. 2016;11(11):e0165925 Epub 2016/11/17. 10.1371/journal.pone.0165925 27851828 PMC5112870

